# Relation between PaO_2_/FiO_2 _ratio, SpO_2_/FiO_2 _ratio, oxygenation index and ventilation ratio in critically ill patients

**DOI:** 10.1186/cc12030

**Published:** 2013-03-19

**Authors:** T Aslanidis, A Myrou, E Chytas, E Anastasiou, E Geka, E Efthimiou, V Ourailoglou, I Soultati, S Primikiri, M Giannakou-Peftoulidou

**Affiliations:** 1 A.H.E.P.A. University Hospital, Thessaloniki, Greece

## Introduction

Many authors have proposed less invasive ways to measure oxygenation in patients with ALI. The aim of the present study is to compare the already popular oxygenation index (OI) and PaO_2_/ FiO_2 _ratio (PFr) with other recently proposed indices such as SpO_2_/FiO_2 _ratio (SFr) and ventilation ratio (VR) in a mixed ICU population.

## Methods

During a 6-month prospective observational study carried out in a polyvalent 10-bed adult ICU, ABGs were obtained from 145 patients. Two independent measurements were taken from each patient under the same mode of ventilation (SIMVPSV). PFr, SFr, VR and OI were calculated. Demographic data (APACHE II score, age, sex, diagnosis, body weight) were also recorded. Kolmogorov-Smirnov test for normality was calculated followed by bivariate nonparametric analysis (statistical significance: *P <*0.05).

## Results

A total of 290 measurements were included for further analysis. Mean ± SD values for age and APACHE II score were 61.2 ± 16 years and 15.4 ± 1.8, respectively, while median ± SD values for PFr are 257 ± 135, for SFr 211 ± 58, for OI 4.8 ± 4.46 and for VR 1.19 ± 0.31. Relations between PFr, SFr and OI are displayed in Table 1.

**Table 1:** Correlations (Spearman ρ) between VR, PFr, SFr and OI

	PFr	SFr	OI
VR	-0.326*	-0.325*	0.373*
PFr	-	0.759*	-0.939*
SFr	-	-	-0.773*
*P <0.01.			

## Conclusion

Our study identified a strong relation between PFr, SFr and OI but not VR. Thus, these markers may be used interchangeably as bedside indices of oxygenation in critically ill patients. Yet larger studies are needed to come to a safer conclusion.
